# Wound closure in epidermolysis bullosa: data from the vehicle arm of the phase 3 ESSENCE Study

**DOI:** 10.1186/s13023-020-01435-3

**Published:** 2020-07-21

**Authors:** Dedee F. Murrell, Amy S. Paller, Christine Bodemer, John Browning, Milos Nikolic, Jay A. Barth, Hjalmar Lagast, Eva Krusinska, Allen Reha, on behalf of the ESSENCE Study Group

**Affiliations:** 1 University of New South Wales, Sydney, NSW Australia; 2 Departments of Dermatology and Pediatric Dermatology, Northwestern University Feinberg School of Medicine, 676 N. St. Clair, Suite 1600, Chicago, IL 60611-2997 USA; 3 EB Expert Centre (MAGEC), Department of Dermatology, Necker-Enfants Malades Hospital, Paris Centre University, Paris, France; 4 Texas Dermatology & Laser Specialists, San Antonio, TX USA; 5 Clinical Center of Serbia, Department of Dermatology, University of Belgrade, Belgrade, Serbia; 6 Amicus Therapeutics, Inc., Cranbury, NJ USA

**Keywords:** Epidermolysis bullosa, Wound closure, Natural history

## Abstract

**Background:**

Chronic wounds are a fundamental issue for patients with epidermolysis bullosa (EB). Herein, we assess the natural history of wound closure in patients with EB who were randomly assigned to the vehicle-control arm of the multicenter, randomized, double-blind, phase 3 ESSENCE (NCT02384460) trial.

**Methods:**

ESSENCE was designed to assess the efficacy and safety of a topical cream formulation of 6% allantoin (SD-101 6%) vs vehicle (SD-101 0%) in patients ≥1 month old who had a diagnosis of EB (simplex, recessive dystrophic, or intermediate junctional) and a target wound 10–50 cm^2^ present for ≥21 days. Time to complete target wound closure and the proportion of patients with target wound closure over time were analyzed overall and by parameters including patient age and baseline body surface area index (BSAi) of total wound burden (< 5% and ≥ 5%). Changes in BSAi of lesional skin, pain, and itching were also assessed.

**Results:**

The vehicle-control arm included 87 patients. Mean (standard deviation [SD]) time to target wound closure within 3 months was 53.6 (28.6) days, with a range of 14 to 142 days. The proportion of patients with target wound closure increased over time from 7.1% at day 14 to 53.6% at month 3. Mean (SD) changes from baseline in BSAi of total wound burden and BSAi of lesional skin at month 3 were −2.3% (6.3) and −5.0% (13.5) of total body coverage, respectively. Reductions in pain and itching were observed at day 7 and maintained for 3 months. Faster healing times and a greater proportion of patients with wound closure were observed in patients aged 1 month to < 2 years; those with wounds < 30 days old, and in those with BSAi of total body wound burden < 5%.

**Conclusions:**

Treatment response observed in the vehicle-control arm of the ESSENCE study was unexpectedly high and may have been due to unforeseen benefits of vehicle or enhanced wound care provided by the clinical trial staff. These observations will help inform the study design of future trials in patients with EB.

**Trial registration:**

ClinicalTrials.gov, NCT02384460; Date of registration: February 13, 2015; First participant enrollment: March 11, 2015.

## Introduction

Epidermolysis bullosa (EB) is a genetically heterogeneous group of rare, devastating disorders characterized by fragility of skin and mucous membranes that blister in response to minor mechanical trauma [1–3]. Signs and symptoms of EB, which include chronic blisters or erosions, ulcers, severe itching, pain, and recurrent wound infection, typically appear in infancy and continue throughout life [4]. Blisters and erosions can occur anywhere on the body, but are typically found in areas of normal mechanical trauma, such as palms, soles, limbs, face, and diaper area [4]. Blisters can also occur in mucous membranes, including the mouth, which can limit food intake and cause scarring with stricture formation [4].

There are 4 main EB types based on the ultrastructural location of skin cleavage: the basal layer of the epidermis for the simplex type, the dermis for the dystrophic type, the epidermal-dermal junction for the junctional type, and multiple cleavage planes for Kindler syndrome [2, 4]. EB simplex is typically the mildest form of the disease and is generally associated with less scarring and fewer internal complications compared with junctional or dystrophic EB [2]. Severe junctional EB (generalized severe sub-type, formerly termed Herlitz sub-type) is associated with the highest risk of infant mortality, usually resulting from sepsis, failure to thrive, or tracheolaryngeal obstruction [2], whereas recessive dystrophic EB is associated with severe scarring and contractures that can substantially decrease physical function [2, 4, 5].

Currently there are no approved specific therapies for EB, and current standard of care involves cleaning and bandaging wounds and pain management [1, 2, 6]. Recommended treatments for wounds can vary depending upon disease severity, ranging from use of nonadherent wound dressings to topical creams and ointments, topical antiseptics, and topical and systemic antibiotics [1]. Wound care standards may also vary between countries or treatment centers, although consensus recommendations, published in 2014, aimed to harmonize and optimize the global management of EB [1].

The phase 3 ESSENCE trial (ClinicalTrials.gov: NCT02384460) was a 3-month, double-blind vehicle-controlled trial designed to assess the efficacy and safety of SD-101, a cream formulation of 6% allantoin (SD-101 6%), in patients with EB [7]. The primary, prespecified results of this study are reported in the companion paper to this article [8]. The inclusion of a vehicle arm (SD-101 with 0% allantoin) in this trial provides an important opportunity to gather data on the natural history of wound closure and other clinical endpoints in patients with EB. This analysis presents descriptive data on baseline wound characteristics and changes from baseline over 3 months of vehicle treatment in the ESSENCE trial, including analyses by patient age, EB type, and baseline wound characteristics (wound age and total body wound burden).

## Methods

### Study design and participants

Detailed methodology of the ESSENCE study is reported in the companion paper to this article [8]. In brief, the ESSENCE study was a multicenter, randomized, double-blind, vehicle-controlled, phase 3 trial designed to assess the efficacy and safety of SD-101 6% vs vehicle (SD-101 0%) in patients with simplex, recessive dystrophic, or intermediate junctional EB. Eligible patients were ≥ 1-month-old with a diagnosis of simplex, recessive dystrophic, or intermediate junctional EB and a target wound between 10 and 50 cm^2^ in size that had been present for ≥21 days (based on patient history). Screening and baseline visits could be combined if patients were eligible. Patients were excluded if they had clinical evidence of local infection in the target wound, had used any investigational drug or systemic or topical steroidal therapy within the 30 days before enrollment (inhaled steroids and ophthalmic drops containing steroids were allowed), had used immunotherapy or cytotoxic chemotherapy within 60 days before enrollment, had used systemic antibiotics within 7 days before enrollment, had current or past malignancy, or had an arterial or venous disorder resulting in ulcerated lesions.

### Treatment

Patients were randomly assigned 1:1 to receive SD-101 6% or vehicle, which was applied topically in a thin layer over the entire body (including all non-wounded areas) once-daily along with daily bandage changes for 90 days using an interactive web response system. SD-101 6% is a topical cream containing 6% allantoin in an oil-in-water emulsion. The vehicle was the same cream formulation as SD-101 but excluding allantoin. SD-101 and the vehicle contained the following excipients: beeswax, butylated hydroxytoluene, cetyl alcohol, citric acid, cod liver oil, lanolin oil, methylparaben, propylene glycol, propylparaben, sodium lauryl sulfate, stearyl alcohol, tetrasodium ethylenediaminetetraacetic acid, and purified water.

### Endpoints and assessments

Primary endpoints were time to complete target wound closure (defined as skin re-epithelialization without drainage) within 3 months and the proportion of patients with target wound closure within 3 months. Key secondary endpoints were proportion of patients with target wound closure (at Months 1 and 2), evaluated using the ARANZ SilhouetteStar^TM^ device; change in body surface area index (BSAi) of lesional skin (percentage of total body coverage of EB-related lesions) and total body wound burden (percentage of total body coverage of open wounds) at Month 3; change from baseline in patient-reported pain using the Face, Legs, Activity, Cry, Consolability (FLACC) scale for patients aged 1 month to 3 years and the Wong-Baker FACES® Pain Scale for patients aged ≥4 years [9, 10] at Day 7; change from baseline in patient-reported itching (via the Itch Man Pruritus Assessment Tool [11]) at Day 7; and the number and incidence of adverse events.

### Statistics

The current analysis includes only data from the vehicle arm of the study, and descriptive results are presented. Demographics, baseline wound characteristics, time to complete wound closure, and the proportion of patients with target wound closure over time were analyzed overall and by patient age (1 month to <2 years, 2 to <12 years, 12 to <18 years, ≥18 years); target wound age (≤30 days and >30 days); baseline BSAi of total wound burden (<5% and ≥5%); and EB type/subtype (simplex, intermediate junctional, and recessive dystrophic). Changes in BSAi of lesional skin, BSAi of total body wound burden, pain, and itching were evaluated over time in the overall vehicle-treated cohort.

## Results

### Patients

Of 169 patients enrolled in the trial, 87 were randomly assigned to the vehicle control group. Eighty patients (92%) in the vehicle control group completed the study; reasons for discontinuation included adverse events (*n =* 2; 2.3%), withdrawal by patient (*n =* 3; 3.4%), and other (*n =* 2; 2.3%; elective bone marrow transplantation [*n =* 1]; could not attend study visits or comply with treatment application [*n =* 1]).

The patient population was heterogeneous and represented a broad range of disease and demographic characteristics (Table 1; individual patient characteristics are presented in Additional file 1). At baseline, target wounds varied considerably in size. The mean (standard deviation [SD]) target wound size in the overall vehicle control group was 22.0 (31.7) cm^2^ and ranged from 7.8 to 302.0 cm^2^. Variations in target wound age were also observed, with patients aged ≥18 years and those with intermediate junctional EB having the most chronic wounds (mean [SD] of 1115 [3350] days and 1676 [3822] days, respectively) (Table 2). Mean (SD) BSAi of total body wound burden was 10.5% (9.1), and most (55 of 87) patients at baseline had a BSAi of total body wound burden ≥5% (Table 2).

**Table 1:** Baseline patient characteristics of the vehicle group

	Total ***n*** = 87	Age Group				Target Wound Age		BSAi of Total Body Wound Burden		EB Type		
		1 month to <2 years ***n*** = 6	2 years to <12 years ***n*** = 47	12 years to <18 years ***n*** = 12	≥18 years ***n*** = 22	≤30 days ***n*** = 25	>30 days ***n*** = 62	<5% ***n*** = 31	≥5% ***n*** = 55	Simplex ***n*** = 8	Recessive dystrophic ***n*** = 62	Intermediate junctional ***n*** = 17
Age, years												
Mean ± SD	13.9 ± 13.1	0.6 ± 0.3	6.8 ± 2.9	14.5 ± 1.8	32.5 ± 12.7	15.5 ± 15.8	13.3 ± 12.0	13.4 ± 15.2	14.0 ± 11.9	6.7 ± 9.1	11.5 ± 9.0	26.4 ± 19.1
Min, Max	0.2, 67.0	0.2, 1.0	2.0, 11.0	12.0, 17.0	18.0, 67.0	0.5, 67.0	0.2, 59.0	0.2, 67.0	0.5, 59.0	0.2, 28.0	0.5, 48.0	2.0, 67.0
Sex, n (%)												
Male	39 (44.8)	3 (50.0)	25 (53.2)	4 (33.3)	7 (31.8)	13 (52.0)	26 (41.9)	15 (48.4)	24 (43.6)	5 (62.5)	28 (45.2)	6 (35.3)
Female	48 (55.2)	3 (50.0)	22 (46.8)	8 (66.7)	15 (68.2)	12 (48.0)	36 (58.1)	16 (51.6)	31 (56.4)	3 (37.5)	34 (54.8)	11 (64.7)
EB Type, n (%)												
Simplex	8 (9.2)	3 (50.0)	4 (8.5)	0	1 (4.5)	1 (4.0)	7 (11.3)	5 (16.1)	3 (5.5)	8 (100)	0	0
Recessive dystrophic	62 (71.3)	3 (50.0)	38 (80.9)	10 (83.3)	11 (50.0)	18 (72.0)	44 (71.0)	23 (74.2)	39 (70.9)	0	62 (100)	0
Intermediate junctional	17 (19.5)	0	5 (10.6)	2 (16.7)	10 (45.5)	6 (24.0)	11 (17.7)	3 (9.7)	13 (23.6)	0	0	17 (100)

*BSAi* Body surface area index, *EB* Epidermolysis bullosa, *SD* Standard deviation

**Table 2:** Baseline wound characteristics of the vehicle group

	Total ***n*** = 87	Age Group				Target Wound Age		BSAi of Total Body Wound Burden		EB Type		
		1 month to <2 years ***n*** = 6	2 years to <12 years ***n*** = 47	12 years to <18 years ***n*** = 12	≥18 years ***n*** = 22	≤30 days ***n*** = 25	>30 days ***n*** = 62	<5% ***n*** = 31	≥5% ***n*** = 55	Simplex ***n*** = 8	Recessive dystrophic ***n*** = 62	Intermediate junctional ***n*** = 17
Target Wound Size, cm^2^												
Mean ± SD	22.0 ± 31.7	15.8 ± 6.4	25.1 ± 42.2	19.3 ± 11.2	18.4 ± 9.5	17.0 ± 9.2	24.0 ± 37.0	14.0 ± 4.7	26.5 ± 39.1	17.7 ± 6.2	23.5 ± 37.1	18.4 ± 11.0
Median	15.5	14.5	16.2	14.4	15.9	12.9	16.3	12.7	17.7	16.1	16.0	12.7
Min, Max	7.8, 302.0	10.0, 27.5	7.8, 302.0	10.0, 45.2	10.1, 45.4	7.8, 43.1	10.0, 302.0	7.8, 25.4	10.1, 302.0	10.1, 27.5	7.8, 302.0	10.0, 45.4
Target Wound Age, days												
Mean ± SD	521.0 ±1832.0	89.0 ±54.8	323.7 ±815.5	420.5 ±1025.2	1115.1 ±3349.7	25.3 ±4.9	720.9 ±2142.6	107.0 ±157.4	763.3 ±2273.5	141.0 ±238.7	253.4 ±656.2	1675.7 ±3821.9
Median	60	87	55	61	59	26	91.5	42	72	59	57.5	83
Min, Max	8, 14,708	21, 1850	21, 4262	21, 3616	8, 14,708	8, 30	31, 14,708	8, 728	21, 14,708	30, 728	21, 3616	8, 14,708
BSAi of Total Body Wound, %												
Mean ± SD	10.5 ± 9.1	5.3 ± 3.9	11.5 ± 10.2	13.2 ± 9.6	8.0 ± 6.2	7.4 ± 6.2	11.7 ± 9.8	3.3 ± 1.3	14.5 ± 9.1	4.6 ± 3.8	11.1 ± 9.9	10.9 ± 6.9
Median	7.6	4.3	8.8	8.7	6.5	4.9	8.1	3.6	11.9	3.3	7.6	8.1
Min, Max	0.8, 47.0	1.9, 12.8	1.0, 47.0	2.8, 30.0	0.8, 25.0	0.8, 25.0	1.1, 47.0	0.8, 4.9	5.3, 47.0	1.2, 12.8	0.8, 47.0	2.8, 25.0

*BSAi* Body surface area index, *EB* Epidermolysis bullosa, *SD* Standard deviation

### Efficacy results in the overall vehicle control group

**Fig. 1:**
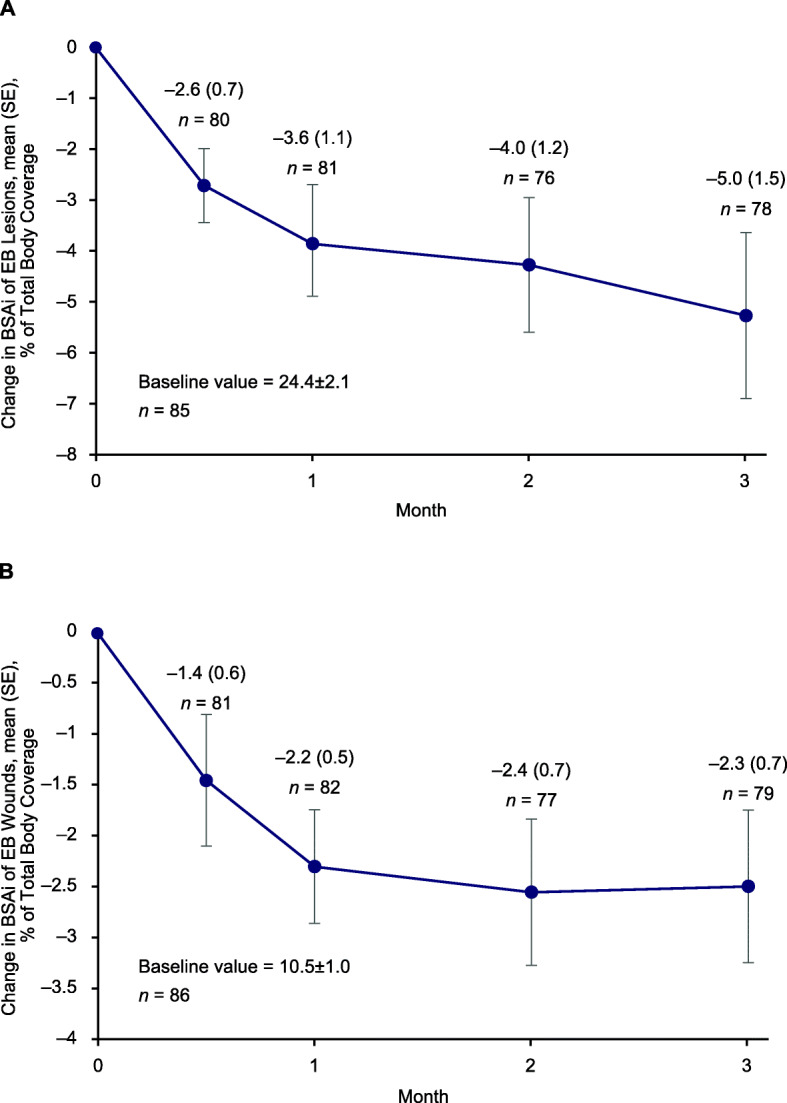
Change in (**a**) lesional skin BSAi, (**b**) total body wound burden BSAi over 3 months. *BSAi* body surface area index; *EB* epidermolysis bullosa; *SE* standard error. Analysis excluded patients with missing values, resulting in variability in patient numbers at each visit

**Fig. 2:**
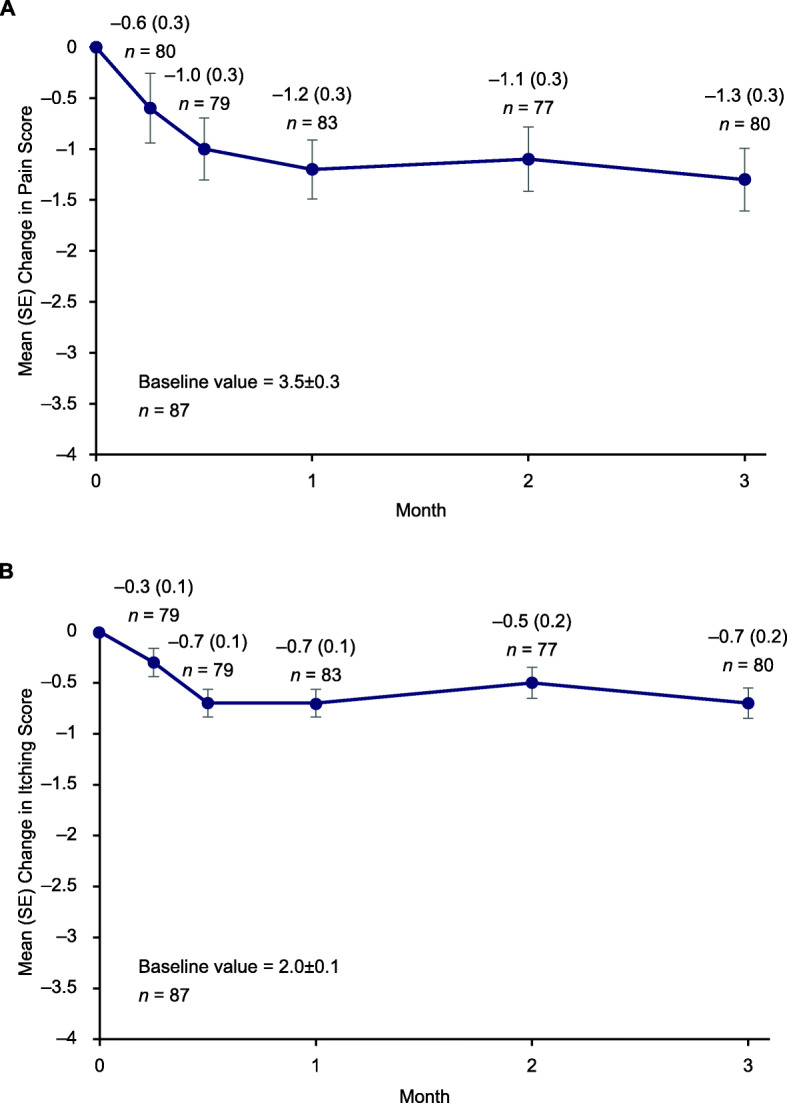
Change in (**a**) pain and (**b**) itch scores over 3 months. *SE* standard error. Analyses excluded patients with missing data

In the overall vehicle control group, mean (SD) time to target wound closure within 3 months was 53.6 (28.6) days, with a median of 57.0 days and a range of 14 to 142 days. The proportion of patients with target wound closure increased over time from 7.1% at day 14 to 53.6% at month 3. Vehicle-treated patients experienced reductions in lesional skin and total body wound burden over the 3-month treatment period. Mean (SD) change from baseline in BSAi of lesional skin at month 3 was −5.0% (13.5) of total body coverage (Fig. 1a). Mean (SD) change from baseline in BSAi of total wound burden at month 3 was −2.3% (6.3) of total body coverage (Fig. 1b). Patients in the vehicle control group also experienced reductions from baseline in pain and itching at the first study visit (Day 7), which were maintained over the 3-month study period (Fig. 2a and b, respectively).

### Subgroup analyses for target wound closure

#### Patient age

**Fig. 3:**
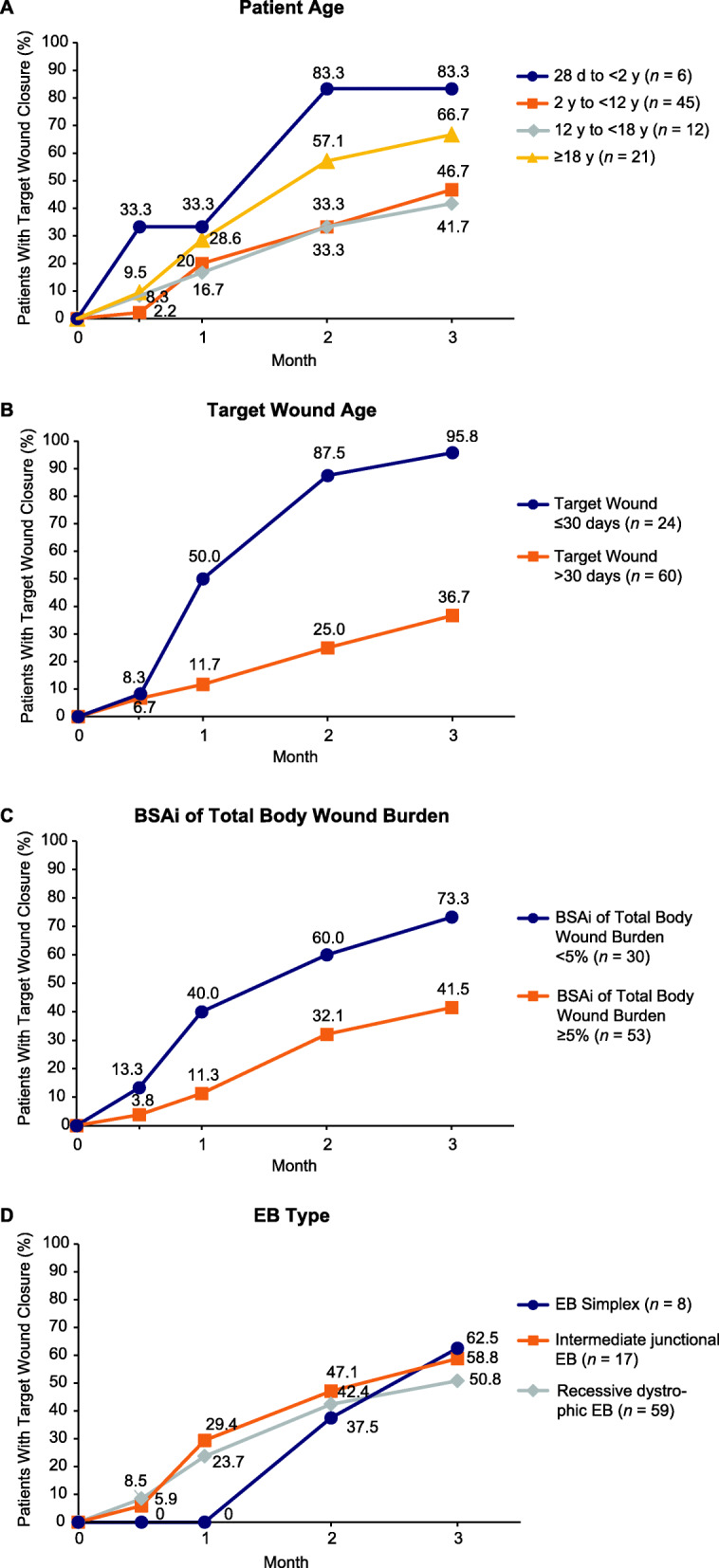
Proportion of vehicle-treated patients with complete target wound closure within 3 months by (**a**) patient age, (**b**) target wound age, (**c**) BSAi of total body wound burden, and (**d**) EB type. *BSAi* body surface area index; *d* day; *EB* epidermolysis bullosa; *y* year

Mean (SD) time to target wound closure was numerically shorter in patients aged 1 month to <2 years (43.8 [23.6] days) compared with patients in older age groups (2 to <12 years, 56.4 [27.8] days; 12 to <18 years, 53.4 [29.1] days; ≥18 years, 52.8 [33.1] days). Differences in the proportions of patients with complete target wound closure over 3 months were also observed by age group, with the youngest age group (1 month to <2 years) having the largest proportion of patients attaining complete target wound closure at month 3 (83.3%) (Fig. 3a).

#### Target wound age

Mean (SD) time to target wound closure was shorter for target wounds ≤30 days old compared with those >30 days old (46.7 [22.2] days vs 60.7 [33.0] days). Twenty-three of 24 (95.8%) evaluable patients with target wounds ≤30 days old achieved target wound closure within 3 months compared with 22 of 60 (36.7%) evaluable patients with target wounds >30 days old (Fig. 3b).

#### BSAi of total wound burden

Mean (SD) time to complete wound closure was shorter in patients with BSAi of total wound burden <5% compared with those with BSAi of total wound burden ≥5% (46.6 [25.4] days vs 60.9 [30.4] days). A higher proportion of patients with BSAi of total wound burden <5% achieved target wound closure within 3 months compared with those who had BSAi of total wound burden ≥5% (73.3% vs 41.5%) (Fig. 3c).

#### EB type

Mean (SD) time to complete wound closure was shorter in patients with recessive dystrophic EB (49.4 [26.2] days) than in those with simplex EB (73.6 [16.6] days) or intermediate junctional EB (56.0 [37.0] days), although the proportion of patients with complete target wound closure was similar at month 3 for simplex and intermediate junctional and slightly lower for recessive dystrophic EB (simplex, 62.5%; intermediate junctional, 58.8%; recessive dystrophic, 50.8%) (Fig. 3d).

## Discussion

To our knowledge, this is the first study to report the natural history of wound closure in patients with EB who applied a vehicle (SD-101 0%) over their *entire* body for 3 month’s duration. The analysis evaluated the most common EB types, as well as patient age, baseline wound age, and baseline total body wound burden. The only other published report of a clinical trial assessing a topical cream in patients with EB in the past 20 years evaluated only the reduction of number of blisters by >40% from baseline in selected areas and in just 17 patients with EB simplex [12]. The present study included 87 patients for the natural history arm, a remarkable sample size for a rare disease.

In the current study, the proportion of patients with each EB type in the vehicle-treated population was similar to those reported in epidemiology studies [13], and most vehicle-treated patients had a baseline BSAi of total wound burden of ≥5% at baseline. In general, wound healing time varied greatly (14 to 142 days), with the youngest patients (1 month to 2 years) having the fastest healing times and the greatest proportion of patients with complete wound closure by 3 months compared with other age groups. When including this young age group in future trials of wound healing in EB or/and other skin diseases, one must consider that rapid healing times may confound the ability to discriminate between active and control treatment arms. A positive aspect of treating young patients is that early healing may lead to fewer complications in this age group. However, due to the small sample size of the youngest age subgroup (*n =* 6), additional natural history data are needed to further understand the extent of differences between this age group versus older patients. In addition, wound closure was observed in a higher proportion of patients and at earlier timepoints in patients with relatively newer wounds (<30 days old) and in those with smaller total wound burden (BSAi <5%). These findings support the importance of patient stratification based on these factors.

It is notable that the proportion of patients with target wound closure within 3 months in vehicle-treated patients did not appear to differ greatly between EB types. These findings are surprising because differences in disease severity have been reported across EB types, with recessive dystrophic EB typically associated with greater disease severity and poorer outcomes compared with patients with EB simplex [2, 4]. It is worth noting that the EB simplex type can be further categorized into subtypes with varying severity [14]. EB simplex subtypes were not reported during the study and potential heterogeneity within EB simplex and the relatively fewer number of patients with EB simplex may have masked the differences between EB simplex and recessive dystrophic EB. Another surprising finding from the current study is that the efficacy response rate in the vehicle arm was much higher than anticipated for natural history, with approximately half of vehicle-treated patients achieving wound closure at 3 months. Natural history studies have shown that patients with recessive dystrophic EB suffer from chronic open wounds that typically last for years [15]. For comparison, the mean time to wound closure in vehicle-treated patients with recessive dystrophic EB in the present study was approximately 7 weeks, with approximately 50% of patients with dystrophic EB achieving wound closure at 3 months.

Several limitations of our study should be considered when interpreting the data. First, there was no statistical comparison between subgroups. Second, patients in this analysis were drawn from a clinical trial that was not designed to evaluate natural history. Third, the number of patients in this study was not balanced across EB types because patients with recessive dystrophic EB most often met the criteria for inclusion in the study. Fourth, daily application of an oil-in-water emulsion cream to the entire body, including areas of nonwounded skin, does not reflect current skin care recommendations for patients with EB [1]. The oil-in-water formulation of the vehicle cream contained excipients including lanolin oil and cod liver oil that may have contributed to the efficacy response observed [16, 17]. Furthermore, daily dressing changes and careful physician monitoring may have contributed to the efficacy response. In the present study, patient visits were typically 3 h in length, and the attention to wound care received by the patients may have been greater than the routine normally practiced at home. In a small, randomized, vehicle-controlled trial of 1% diacerein topical cream in patients with EB simplex conducted by Wally and colleagues, the authors attributed a small placebo effect in the control arm to regular and intensive wound care received during the study period [12]. Because at-home wound care routines and the frequency of physician visits before study initiation were not collected in sufficient detail, we cannot determine how trial-related activities may have influenced efficacy assessments in the vehicle control group. In addition, patient expectations and beliefs about a potential treatment and the possibility of a beneficial outcome can also cause a “placebo effect,” an observation that is becoming increasingly apparent in dermatology [18]. Given the significant burden of EB, and the lack of an approved therapy, it is reasonable to assume that both patient and caregiver’s expectations of treatment in the present study was high. Finally, there was no control group that included patients who practiced standard of care or their “usual” wound care routine that could be compared to the vehicle-control group.

## Conclusions

Treatment response observed in the vehicle-control arm of the ESSENCE study was unexpectedly high, with overall improvements observed in target wound closure, lesional skin and total body wound burden, itch, and pain over the 3-month treatment period. The reason for these improvements could be due to several factors, including unforeseen beneficial effects of the oil-in-water formulation or other excipients in the topical cream, as well as enhanced wound care provided by clinical trial staff. Patients aged <2 years, patients with target wounds ≤30 days old, and patients with BSAi of total wound burden <5% tended to have shorter time to target wound closure and greater wound closure rate within 3 months while receiving vehicle treatment. These observations are of considerable interest and may help inform the study design of future trials in patients with EB, which may benefit from a vehicle run-in phase to exclude patients who respond to vehicle controls or improved wound care.

At present, wound care in patients with EB should conform to current treatment guidelines, with consistency across treatment centers, to optimize management of this condition. However, topical application of an emollient cream to both wounded and nonwounded skin, especially with frequent dressing changes, may encourage barrier repair in patients with EB. Such a management strategy may reduce itch and further trauma to existing wounds, preserve nonwounded skin and, hence, prove useful in the management of patients with EB.

## Supplementary information

**Additional file 1.** ESSENCE individual baseline characteristics of the vehicle group. Contains individual de-identified data of patients who were randomly assigned to the vehicle group in the current study.

## Data Availability

The data supporting the findings of this study are reported in the companion manuscript (entitled “Efficacy and tolerability of the investigational topical cream SD-101 [6% allantoin] in patients with epidermolysis bullosa: a phase 3, randomized, double-blind, vehicle-controlled trial [ESSENCE Study])” and its additional file, which was submitted contemporaneously to *Orphanet Journal of Rare Diseases*.

## Abbreviations

BSAi: Body surface area index
EB: Epidermolysis bullosa
FLACC: Face, Legs, Activity, Cry, Consolability scale
SD: Standard deviation
SE: Standard error
